# Supplementary material for: Efficacy of a Covalent Microtubule Stabilizer in Taxane-Resistant Ovarian Cancer Models
Source: Molecules. 2021 Jul 3;26(13):4077. doi: 10.3390/molecules26134077 (PMC8271594; doi:10.3390/molecules26134077)
Supplement: Supplementary file 1 [file molecules-26-04077-s001.zip › molecules-1239075-supplementary.pdf]

Supplementary Materials

Efficacy of a Covalent Microtubule Stabilizer in Taxane-Resistant Ovarian Cancer Models

Samantha S. Yee [1,2] and April L. Risinger [1,2,*]

[1] Department of Pharmacology, The University of Texas Health Science Center at San Antonio, Floyd Curl Drive, San Antonio, TX 78229, USA; yees3@livemail.uthscsa.edu
[2] Mays Cancer Center, 7979 Wurzbach Road, San Antonio, TX 78229, USA
* Correspondence: risingera@uthscsa.edu; Tel.: +1-210-567-6267

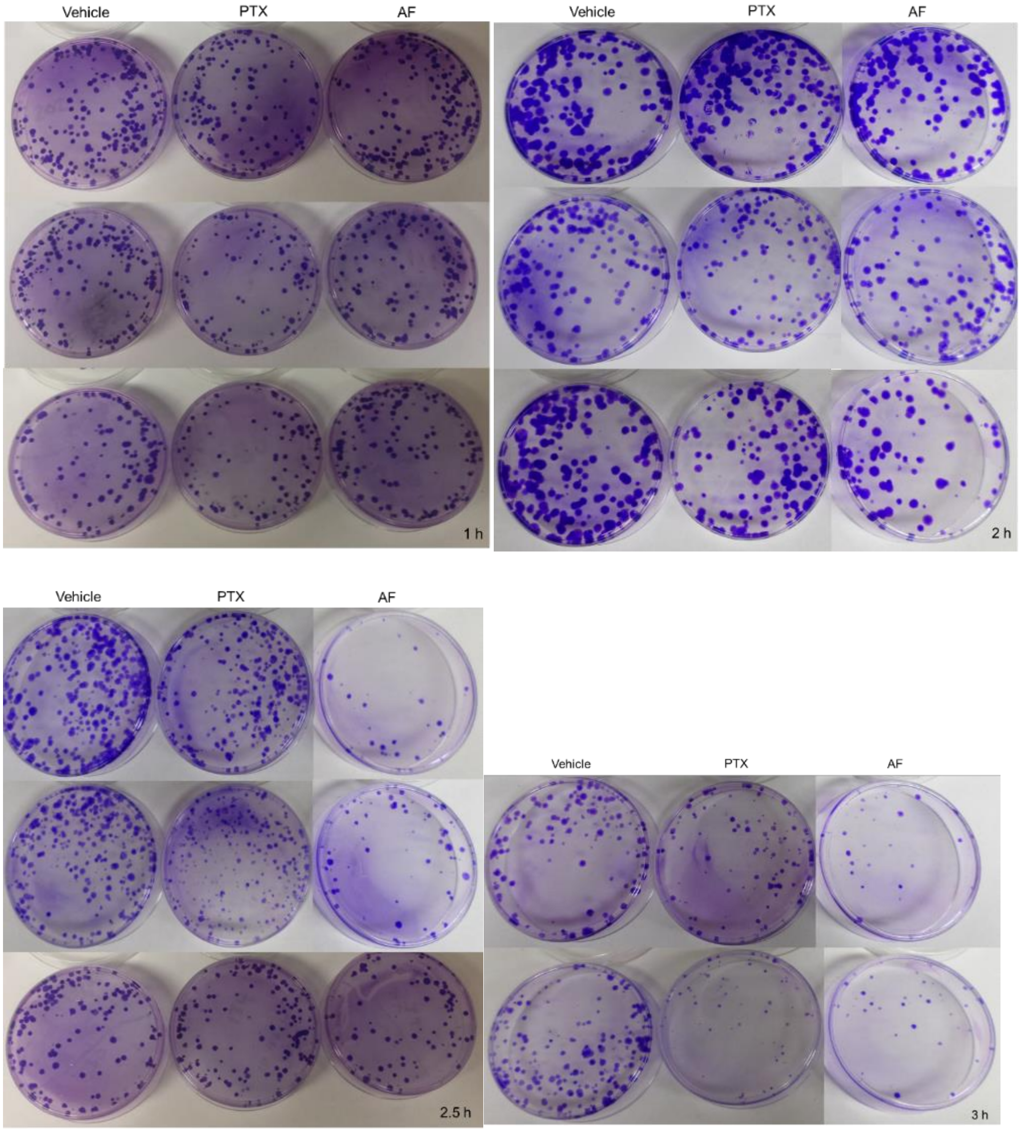

Figure S1. The taccalonolides have potent and persistent effects in the SK-OV-3 human ovarian cancer cell line following acute exposure for 1–3 h with 50 nM PTX, taccalonolide AF or vehicle followed by drug wash-out and replacement with fresh media. Colonies grew for 12 days after drug was removed (n = 2–3 independent experiments). Images of each of the persistence assay plates are shown for all the different timepoints.

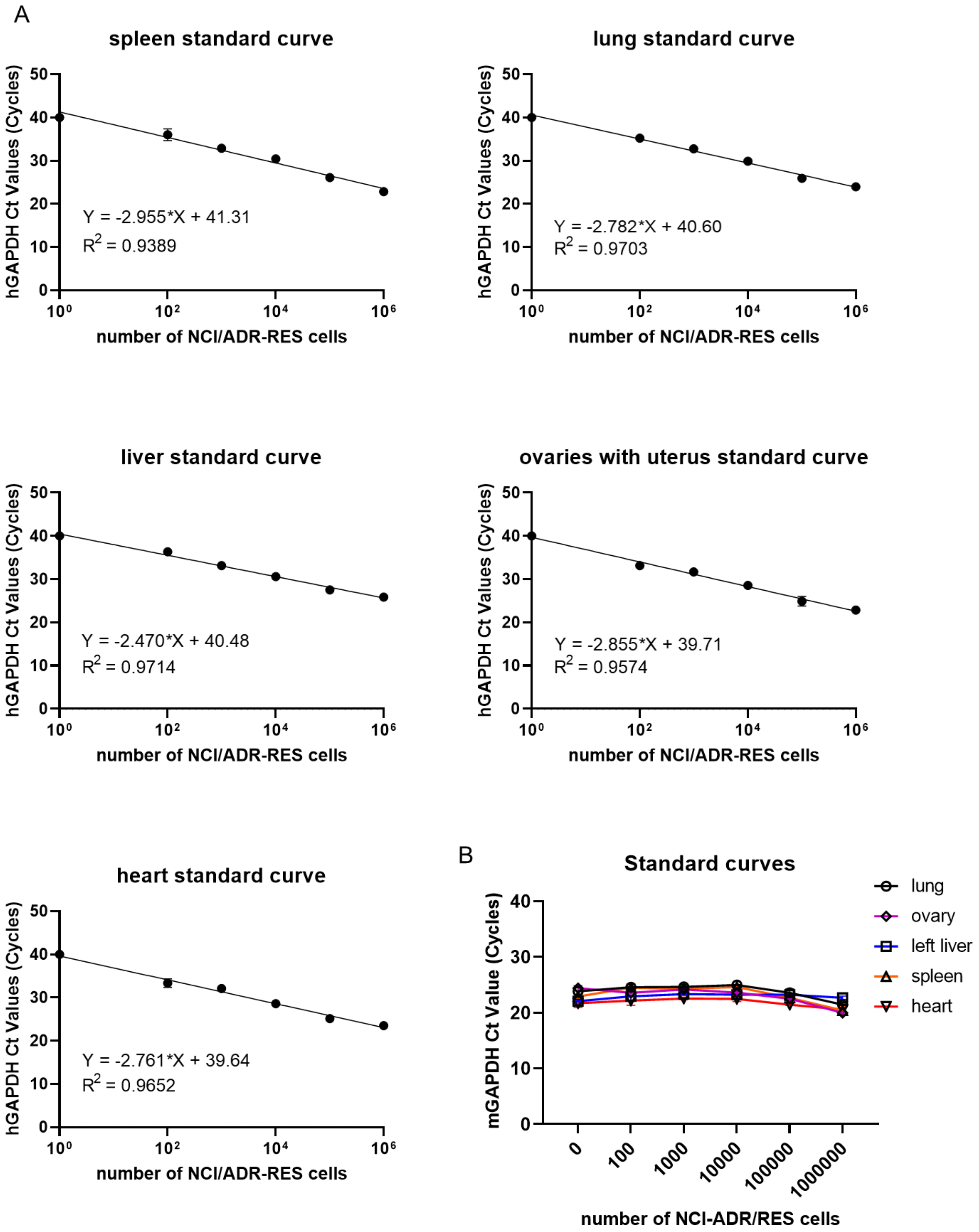

Figure S2. Standard curves to detect the number of NCI/ADR-RES cells in mouse organs to be used in quantification of a disseminated metastatic ovarian cancer murine model. (**A**) Standard curves of NCI/ADR-RES cells in indicated mouse tissues as determined by quantifying the human GAPDH Ct values when a known number of human NCI/ADR-RES cells were spiked into naive mouse organs for the spleen, lung, liver, ovaries with uterus, and heart. (**B**) Quantification of murine GAPDH values when a known number of human NCI/ADR-RES cells were spiked into naive mouse organs for the spleen, lung, liver, ovaries with uterus, and heart. n = 2–3 independent experiments were conducted to generate the standard curves.

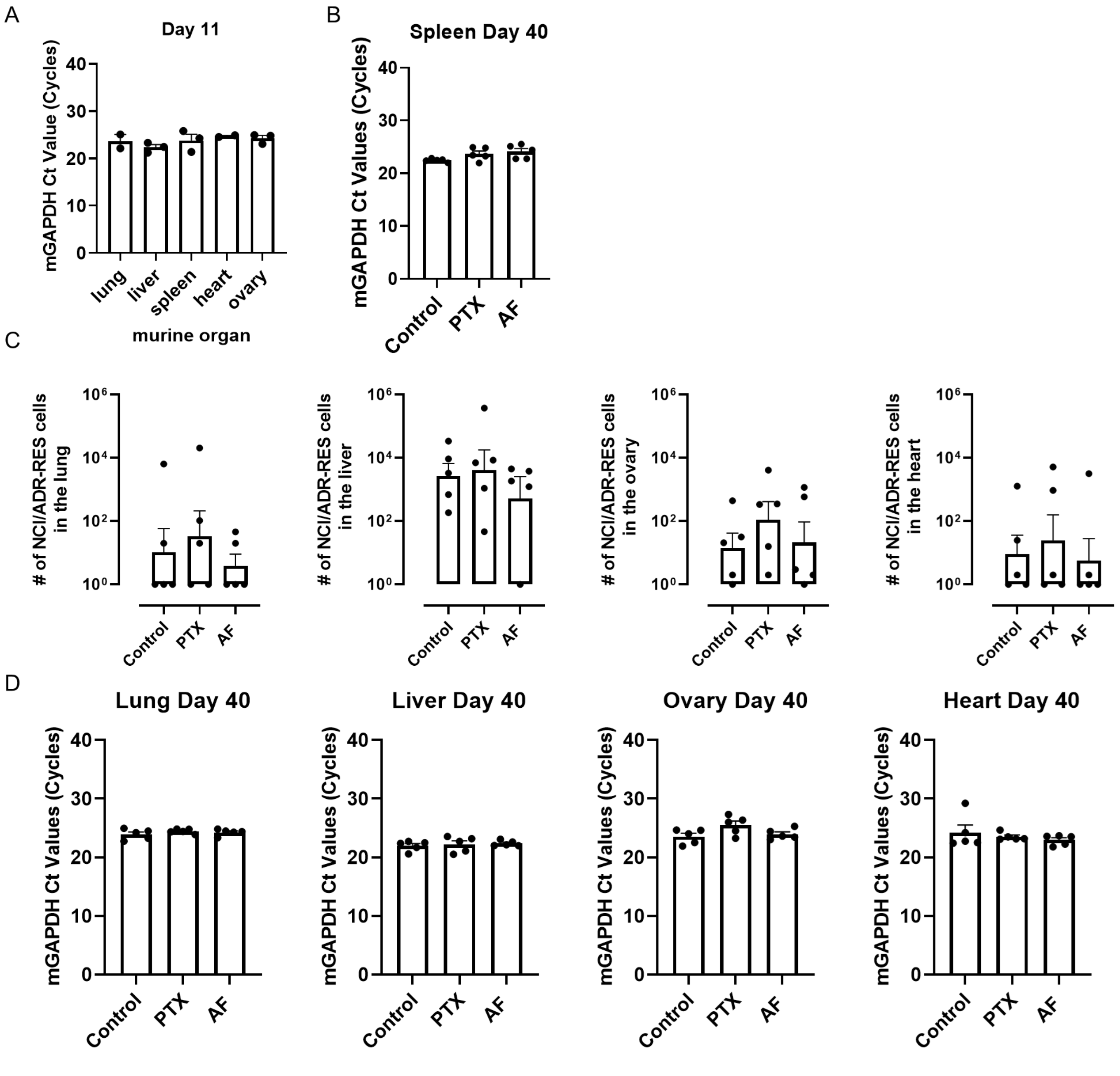

Figure S3. Quantification of i.p. disseminated NCI/ADR-RES cells in mouse tissues by qRT-PCR. (**A**) Quantification of murine GAPDH from tissues harvested 11 days after NCI/ADR-RES cells were injected (n = 2–3 independent experiments). (**B**) Quantification of murine GAPDH from spleens harvested 40 days after NCI/ADR-RES cells were injected in the presence of indicated drug treatment (n = 5). (**C**) Quantification of human NCI/ADR-RES cells in mouse tissues harvested 40 days after NCI/ADR-RES cells were injected in the presence of indicated drug treatment (n = 5). (**D**) Quantification of murine GAPDH in mouse from tissues harvested 40 days after NCI/ADR-RES cells were injected in the presence of indicated drug treatment (n = 5). Closed circles represent individual data points

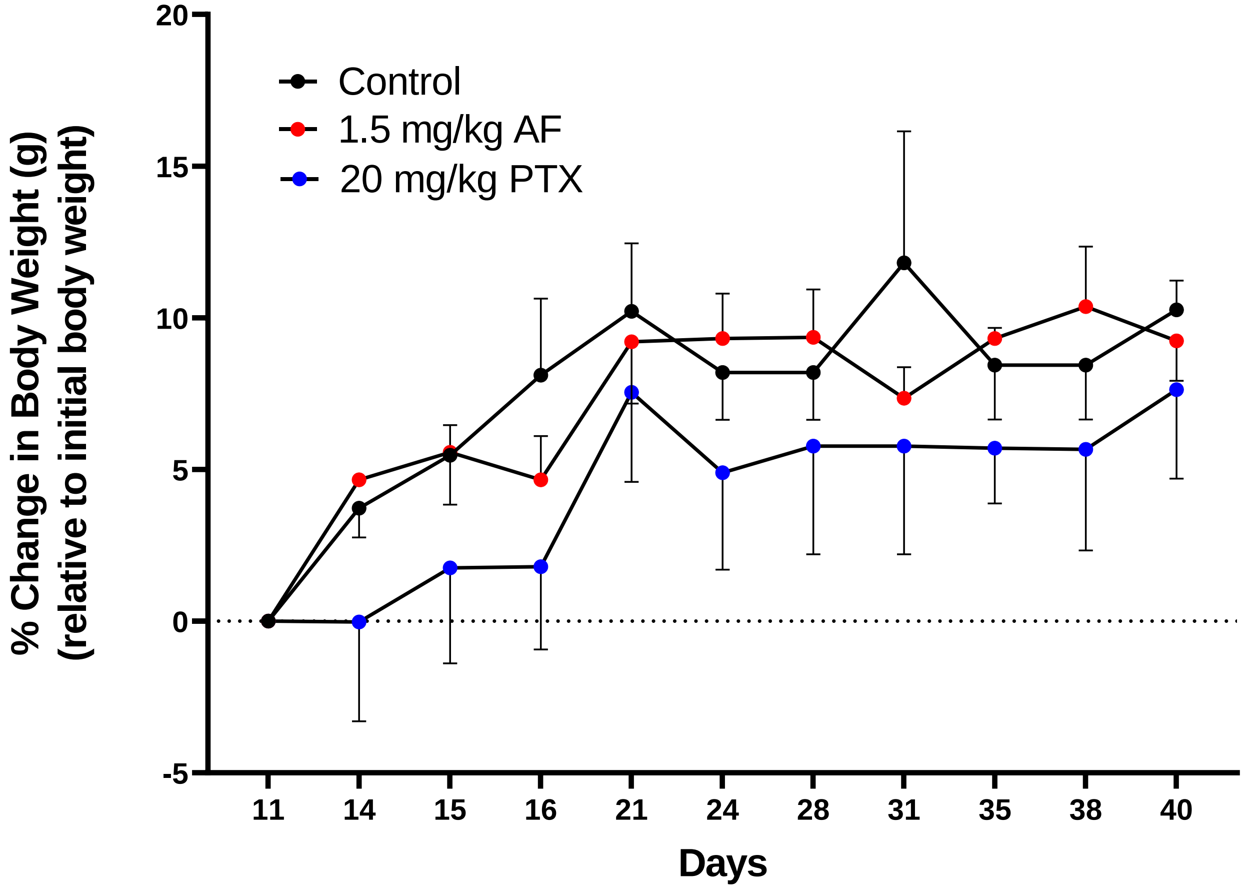

Figure S4. Percent change in bodyweight for animals bearing i.p. disseminated NCI/ADR-RES cells during the course of treatment with 1.5 mg/kg AF, 20 mg/kg PTX, or vehicle on days 11 and 14 (n = 5 mice/treatment group).
